# Diagnostic Discordance in a 46,XX Ovarian Dissecting Gonadoblastoma with Dysgerminoma Transformation and Leydig Cell Tumor: Hyperandrogenism, β-hCG Elevation, and CEUS Findings

**DOI:** 10.3390/diagnostics16193223

**Published:** 2026-10-05

**Authors:** Jiahui Yu, Jiang Zhu

**Affiliations:** 1 Department of Ultrasound, Women’s Hospital, Zhejiang University School of Medicine, Hangzhou 310006, China; 15857979508@163.com; 2 Zhejiang Provincial Key Laboratory of Precision Diagnosis and Therapy for Major Gynecological Diseases, Women’s Hospital, Zhejiang University School of Medicine, Hangzhou 310006, China

**Keywords:** dissecting gonadoblastoma, dysgerminoma, Leydig cell tumor, germ cell–sex cord-stromal tumor, hyperandrogenism, β-hCG, contrast-enhanced ultrasound, 46,XX

## Abstract

**Background and Clinical Significance:** The current WHO classification recognizes gonadoblastoma and mixed germ cell–sex cord-stromal tumor, unclassified, as distinct ovarian germ cell–sex cord-stromal tumors. Their heterogeneous components may generate discordant endocrine, biochemical, and imaging findings. **Case Presentation:** An 18-year-old nulliparous woman with a 46,XX peripheral blood karyotype presented with progressive virilization and an enlarging left ovarian mass. Preoperative total testosterone was >16,000 pg/mL and β-human chorionic gonadotropin (β-hCG) was 192.0 IU/L. Magnetic resonance imaging (MRI) and transrectal contrast-enhanced ultrasound (CEUS) favored an androgen-producing sex cord-stromal tumor; CEUS showed heterogeneous hyperperfusion with mildly disorganized intratumoral vessels. Computed tomography (CT) suggested a mixed germ cell tumor, and frozen section favored a steroid cell tumor while not excluding a focal germ-cell component. Fertility-sparing surgery was performed. Final pathology showed dissecting gonadoblastoma with dysgerminoma transformation and trophoblastic differentiation, combined with a Leydig cell tumor. Serum β-hCG fell to 80.4 IU/L on postoperative day 1 and to <0.2 IU/L by January 2026, accompanied by androgen normalization and resumption of menstruation. At 6.5 months, the patient remained clinically well; ultrasound showed a normal right ovary and no evident left adnexal mass, although contemporaneous endocrine and tumor-marker measurements were unavailable. **Conclusions:** In a heterogeneous ovarian tumor, the dominant phenotype may represent only one component. Discordance among endocrine findings, tumor markers, and imaging should prompt consideration of mixed histology. β-hCG elevation in an androgen-secreting ovarian tumor may indicate an occult germ-cell or trophoblastic component, whereas CEUS should be interpreted as a vascular phenotype rather than a histology-specific signature.

## 1. Introduction

The current WHO classification recognizes gonadoblastoma and mixed germ cell–sex cord-stromal tumor, unclassified, as distinct entities within ovarian germ cell–sex cord-stromal tumors [1]. Gonadoblastoma comprises germ cells and sex cord elements and classically develops in dysgenetic gonads, usually in association with Y-chromosome material [2,3]. Its dissecting variant may obscure the classic nested architecture and can mimic invasive germinoma/dysgerminoma [3,4]. By contrast, mixed germ cell–sex cord-stromal tumor, unclassified, is a separate entity and should not be used synonymously with gonadoblastoma. Nevertheless, gonadoblastoma has been documented in phenotypically normal individuals with a 46,XX peripheral blood karyotype [5,6,7,8].

The coexistence of germ-cell and sex cord-stromal components creates a particular diagnostic challenge. A hormonally active steroid-producing sex cord-stromal component may dominate the clinical picture through marked androgen excess and virilization [9], whereas a coexisting germ-cell or trophoblastic component may contribute tumor-marker abnormalities or distinct imaging features. In such cases, apparently discordant findings may not represent diagnostic error; instead, they may reflect different biological components of the same tumor.

Ultrasound and CEUS can characterize ovarian morphology and microvascular perfusion, but vascularity is not specific to one histologic category. Ovarian dysgerminomas are often highly vascularized on Doppler ultrasound [10], and sex cord-stromal tumors can also demonstrate marked enhancement on CEUS [11]. A recent CEUS case report of an ovarian Sertoli–Leydig cell tumor further illustrates substantial vascular overlap among endocrine-active ovarian tumors [12].

We report an 18-year-old phenotypically normal woman with a 46,XX karyotype who presented with profound hyperandrogenism, elevated β-hCG, and a rapidly enlarging hypervascular ovarian mass. The endocrine phenotype, magnetic resonance imaging (MRI), and CEUS were interpreted as favoring an androgen-producing sex cord-stromal tumor, whereas computed tomography (CT) raised the possibility of a mixed germ cell tumor. Frozen section favored a steroid cell tumor but specifically did not exclude a focal germ-cell component. Final pathology unified these findings by demonstrating dissecting gonadoblastoma with dysgerminoma transformation and trophoblastic differentiation, combined with a Leydig cell tumor. The aim of this report is to highlight diagnostic discordance as a clue to mixed histology and to clarify the role—and limitations—of CEUS in this setting.

## 2. Case Presentation

### 2.1. Clinical Course and Endocrine Findings

An 18-year-old nulliparous Chinese woman with no history of sexual activity was admitted in December 2025 for menstrual irregularity, progressive virilization, and an enlarging left adnexal mass. She had previously had regular menses but developed prolonged spotting in 2022. Combined oral contraceptives were prescribed intermittently, with only temporary control of bleeding.

In 2025, the clinical course became more aggressive. Acne and deepening of the voice became prominent from approximately June. On 2 September 2025, serum testosterone was 18.7 nmol/L, and ultrasonography at that visit showed a 4.1 × 3.8 × 2.9 cm cystic lesion in the left ovary. Repeat ultrasonography on 12 October showed a 3.8 × 2.8 × 2.8 cm left ovarian cystic lesion. By 23 October, testosterone had risen to 42 nmol/L despite continued hormonal treatment. On 19 December, testosterone exceeded 52 nmol/L, and transrectal ultrasound demonstrated an 8.9 × 7.1 × 8.5 cm cystic-solid left adnexal mass. The patient had also developed secondary amenorrhea, intermittent lower abdominal discomfort, and a 6 kg weight gain over approximately 2 months.

On examination, multiple acneiform lesions involved the face, neck, chest, back, buttocks, and thighs. The external genitalia were phenotypically female without an obvious developmental abnormality. No relevant family history of gonadal tumors or inherited endocrine disease was recorded.

Preoperative endocrine testing showed profound androgen excess: total testosterone > 16,000 pg/mL, androstenedione > 10,000 pg/mL, and 17α-hydroxyprogesterone > 20,000 pg/mL. Dehydroepiandrosterone (DHEA) was 3698.95 pg/mL and dehydroepiandrosterone sulfate (DHEAS) was 2093 ng/mL. Luteinizing hormone (LH) and follicle-stimulating hormone (FSH) were both <0.30 IU/L. Serum β-hCG was 192.0 IU/L despite the absence of pregnancy. This marker abnormality was discordant with the leading diagnosis of a pure androgen-producing sex cord-stromal tumor. Given the extreme androgen elevation, the available inpatient evaluation also included serum cortisol and adrenocorticotropic hormone (ACTH), which showed no obvious abnormality. An ovarian source was strongly favored preoperatively because the progressive rise in testosterone paralleled rapid enlargement of a unilateral ovarian mass; however, a dedicated dynamic adrenal/congenital adrenal hyperplasia work-up or selective venous sampling was not documented. The rapid postoperative decline in testosterone and other androgen precursors after left salpingo-oophorectomy subsequently provided strong biochemical support for a tumor-related ovarian source.

### 2.2. Multimodal Imaging

Contrast-enhanced pelvic MRI on 23 December 2025 showed an 8.3 × 6.9 × 8.1 cm predominantly solid cystic-solid mass, with marked enhancement of the solid component. In the context of severe hyperandrogenism, a left ovarian sex cord-stromal tumor was favored.

Transrectal three-dimensional ultrasonography on 24 December demonstrated a well-circumscribed 9.2 × 9.4 × 6.3 cm cystic-solid mass. The cystic component measured approximately 5.0 × 4.7 × 3.9 cm and contained clear fluid. The solid component showed intermediate-to-high echogenicity with focal microcystic changes and internal vascular signals. CEUS demonstrated heterogeneous hyperperfusion with rich and mildly disorganized intratumoral vessels. Based on the endocrine phenotype and vascular imaging appearance, the ultrasound diagnosis favored a Leydig cell tumor or another androgen-producing sex cord-stromal tumor.

The preoperative imaging assessment was not uniform. Contrast-enhanced abdominal and pelvic CT demonstrated an approximately 10.1 × 6.4 cm cystic-solid pelvic mass containing multiple enhancing solid nodular and mass-like components, the largest measuring approximately 6.2 × 5.0 cm. The adjacent mesenteric structures were blurred and the left ovary was poorly visualized; no enlarged pelvic or para-aortic lymph nodes or obvious peritoneal nodules were reported. On the basis of this heterogeneous morphology, the interpreting radiologist raised the possibility of a mixed ovarian germ cell tumor. Thus, MRI and CEUS were interpreted as favoring an androgen-producing sex cord-stromal tumor, whereas CT raised suspicion for an additional germ-cell component. The multimodal imaging findings are shown in Figure 1.

### 2.3. Surgery and Pathological Diagnosis

On 24 December 2025, the patient underwent laparoscopic left salpingo-oophorectomy, multiple peritoneal biopsies, partial omentectomy, and pelvic adhesiolysis as a fertility-sparing staging procedure. Approximately 150 mL of clear yellow pelvic fluid was noted. The left ovary measured approximately 9.0 × 8.0 × 7.5 cm and was cystic-solid and enlarged. The right ovary was grossly free of tumor. No obvious peritoneal, omental, upper abdominal, or enlarged pelvic/para-aortic nodal lesions were identified. Cytological examination of the peritoneal fluid was negative for malignant cells.

The resected ovary was multilocular and cystic-solid, with a friable brown solid component. Intraoperative frozen section favored a steroid cell tumor but specifically noted that a focal germ-cell tumor component could not be excluded.

Histopathologic examination demonstrated a heterogeneous tumor with multiple gonadoblastoma patterns. Classic gonadoblastoma nests composed of admixed germ cells and sex cord elements were identified, whereas other areas showed a dissecting/irregular growth pattern. Foci with relative predominance of sex cord-stromal elements were also present within the gonadoblastoma component. In addition, a distinct Leydig cell tumor component was identified, characterized by polygonal cells with abundant eosinophilic cytoplasm. The complete histopathologic evaluation of the resection specimen established the diagnosis of dissecting gonadoblastoma with dysgerminoma transformation and trophoblastic differentiation, together with a Leydig cell tumor component. The institutional pathology report used the broader label “mixed germ cell–sex cord-stromal tumor.” The laparoscopic specimen was fragmented, limiting precise pathological measurement of tumor size; clinical and imaging correlation was therefore recommended.

Immunohistochemistry supported the composite nature of the tumor. FOXL2, Calretinin, Inhibin, and SF1 were positive, supporting sex cord-stromal differentiation, whereas CD117/c-KIT, OCT4, PLAP, and SALL4 supported germ-cell differentiation. HCG was positive in a small number of cells, concordant with the reported trophoblastic differentiation. Glypican-3 was also positive in a small number of cells, whereas AFP was negative. Fluorescence in situ hybridization (FISH) for i(12p) was negative. Peripheral blood karyotyping showed 46,XX. Representative hematoxylin and eosin-stained sections are shown in Figure 2.

### 2.4. Postoperative Course, Genetic Counseling, and Follow-Up

The biochemical response to tumor removal was rapid. Serum β-hCG decreased from 192.0 IU/L preoperatively to 80.4 IU/L on postoperative day 1 and to <0.2 IU/L by January 2026. Testosterone decreased to 28.1 nmol/L on postoperative day 1 and subsequently to 104.7 pg/mL. Androstenedione decreased to 638.2 pg/mL and 17α-hydroxyprogesterone to 156.8 pg/mL. Spontaneous menstruation resumed shortly after surgery.

The postoperative clinical diagnosis was recorded as stage IA ovarian malignancy. At outpatient multidisciplinary team (MDT) review, preservation of the contralateral ovary with close surveillance was considered acceptable in view of the patient’s young age, nulliparity, 46,XX peripheral blood karyotype, and the absence of a grossly evident tumor in the contralateral ovary. The MDT also documented concern about adverse prognostic features and discussed the possibility of four cycles of adjuvant chemotherapy. However, the contemporaneous MDT record did not itemize which individual pathological features were regarded as adverse and did not specify a proposed chemotherapy regimen. Potential chemotherapy-related toxicities, including bone-marrow suppression, allergic reactions, ovarian dysfunction, and hepatic or renal dysfunction, were discussed. The family initially elected to consider the options before deciding whether to proceed. The patient and her family subsequently elected not to receive chemotherapy, and no adjuvant treatment was administered.

Because gonadoblastoma in a 46,XX patient is unusual, genetic consultation was obtained on 17 January 2026. Whole-exome sequencing was not recommended, and its limitations were explained. No tumor-specific testing for Y-derived material, such as SRY FISH, Y-chromosome short tandem repeat analysis, or TSPY testing, was documented.

At 6.5 months after surgery, the patient remained clinically well with sustained spontaneous menstruation; the last recorded menstrual period began on 30 June 2026 and was of moderate volume. Transrectal three-dimensional ultrasonography on 7 July 2026 showed a normal uterus and right ovary, and no evident mass was identified in the left adnexal region. Contemporaneous endocrine and tumor-marker results were not available in the reviewed medical record. The longitudinal clinical, biochemical, and imaging course is summarized in Table 1. A completed CARE checklist is provided as Supplementary File S1.

## 3. Discussion

### 3.1. Diagnostic Discordance as a Clue to Mixed Histology

The principal educational value of this case is that apparently discordant findings mapped onto different tumor components. Rapidly progressive virilization, extreme testosterone elevation, and a hypervascular ovarian mass strongly suggested an androgen-producing sex cord-stromal tumor, particularly a steroid cell or Leydig cell tumor, an interpretation consistent with the known hormonal activity of ovarian steroid cell tumors [9].

However, a single-histology diagnosis did not explain the entire picture. Serum β-hCG was 192.0 IU/L; CT raised the possibility of a mixed germ cell tumor; and frozen section favored a steroid cell tumor while specifically cautioning that a focal germ-cell component could not be excluded. Permanent pathology reconciled these findings by demonstrating dissecting gonadoblastoma with dysgerminoma transformation and trophoblastic differentiation, together with a Leydig cell tumor in the same ovary.

This case therefore supports a practical diagnostic principle: when endocrine manifestations, tumor markers, and imaging findings cannot all be explained by one favored ovarian tumor category, the discordance itself may be biologically meaningful. Mixed histology should be considered rather than forcing all findings into the dominant phenotype. The major discordant findings and their final clinicopathological interpretation are summarized in Table 2.

### 3.2. What CEUS Demonstrated—And What It Could Not Determine

CEUS demonstrated heterogeneous hyperperfusion and a rich, mildly disorganized vascular network. These findings contributed to the preoperative impression of an androgen-producing sex cord-stromal tumor. However, marked vascularity is not specific. Ovarian dysgerminomas are commonly moderately to very well vascularized on Doppler ultrasound [10], and CEUS studies of sex cord-stromal tumors have also described hyperenhancement, particularly in non-benign lesions [11]. A published CEUS case of an ovarian Sertoli–Leydig cell tumor similarly showed that endocrine-active sex cord-stromal tumors may be strikingly vascular [12].

Accordingly, CEUS in this case was valuable for phenotyping tumor perfusion but not for defining the complete histology. In a mixed neoplasm, the dominant or most vascular component may disproportionately shape the enhancement pattern. The observed CEUS phenotype therefore did not exclude coexisting gonadoblastoma, dysgerminoma, or trophoblastic differentiation.

Focal microcystic changes were visible within the solid component on conventional ultrasound. The available pathological material did not establish a direct histological correlate for these small spaces. We therefore regard them as a descriptive imaging feature rather than a specific sign of dysgerminoma-associated ischemia, necrosis, or malignant transformation.

### 3.3. β-hCG as a Discordant Biochemical Signal

The elevated β-hCG was the clearest biochemical finding that did not fit a pure androgen-producing tumor. Dysgerminomas may occasionally contain syncytiotrophoblastic giant cells; a large modern pathological series confirmed this finding in a minority of cases [13]. Importantly, a prior report described HCG positivity in a 15-year-old patient with gonadoblastoma and dysgerminoma despite a 46,XX karyotype [7]. Thus, β-hCG elevation in this setting is not unprecedented and should not be presented as a unique feature.

What is particularly instructive in the present case is the clinicopathological correlation between two functional tumor signals. Serum β-hCG was elevated preoperatively; the final pathological diagnosis included trophoblastic differentiation; HCG positivity was present in a small number of cells; and β-hCG declined rapidly after resection. In parallel, the pathologically confirmed Leydig cell tumor component provided a coherent explanation for the profound hyperandrogenism. The two biochemical abnormalities therefore represented different functional components within the same heterogeneous tumor.

For clinical practice, an otherwise unexplained β-hCG elevation in an androgen-secreting ovarian tumor should prompt deliberate consideration of an additional germ-cell or trophoblastic component, even when MRI or CEUS strongly favors a sex cord-stromal tumor.

### 3.4. 46,XX Gonadoblastoma and Pathological Context

The current WHO classification treats gonadoblastoma and mixed germ cell–sex cord-stromal tumor, unclassified, as separate entities within ovarian germ cell–sex cord-stromal tumors [1]. This distinction is particularly relevant in phenotypically normal patients without an evident disorder of sex development, in whom gonadoblastoma may be confused with mixed germ cell–sex cord-stromal tumor, unclassified [14]. The institutional pathology report used the broader label “mixed germ cell–sex cord-stromal tumor” but specified dissecting gonadoblastoma with dysgerminoma transformation and trophoblastic differentiation, combined with a Leydig cell tumor. We therefore use the component-level diagnosis throughout the revised manuscript rather than treating MGC-SCST as a synonym for gonadoblastoma. Classic gonadoblastoma is strongly associated with dysgenetic gonads and Y-chromosome material [2,3], but well-documented cases have occurred in phenotypically normal individuals with a 46,XX peripheral karyotype [5,6,7,8].

The diagnosis of dissecting gonadoblastoma is also relevant because this pattern can mimic invasive germinoma/dysgerminoma morphologically; integration of architecture and immunohistochemistry is essential [3,4]. In the present case, the component-level diagnosis was rendered after complete histopathological and immunohistochemical evaluation. FOXL2, Calretinin, Inhibin, and SF1 supported sex cord-stromal differentiation, whereas OCT4, SALL4, PLAP, and CD117/c-KIT supported germ-cell differentiation [15]. HCG positivity in a small number of cells was concordant with the reported trophoblastic differentiation; Glypican-3 was also positive in a small number of cells, whereas AFP was negative.

Peripheral blood karyotyping demonstrated 46,XX, and FISH for i(12p) was negative. However, i(12p) testing does not assess Y-derived material, and no tumor-specific testing for Y-chromosome sequences was documented. Therefore, very-low-level Y-chromosome mosaicism cannot be completely excluded, and this limitation should be considered when interpreting the genetic context of the tumor.

### 3.5. Fertility Preservation and Postoperative Uncertainty

Fertility preservation was a major clinical consideration because the patient was 18 years old and no grossly evident tumor was identified in the contralateral ovary at surgery. Current international guidance provides recommendations on fertility-sparing treatment and follow-up for selected patients with ovarian cancer [16].

The postoperative MDT considered preservation of the contralateral ovary with close surveillance acceptable, but also discussed four cycles of adjuvant chemotherapy because of concern about adverse prognostic features. Importantly, the contemporaneous MDT record did not specify which individual pathological findings were considered adverse or document a proposed chemotherapy regimen. Chemotherapy was therefore discussed as a possible option rather than formally prescribed, and the patient and her family ultimately elected not to receive adjuvant treatment. This individualized course reflects the uncertainty surrounding an exceptionally rare mixed tumor and should not be interpreted as evidence that surveillance alone is a standard strategy for this entity.

At 6.5 months after surgery, spontaneous menstruation was maintained, the right ovary appeared normal on ultrasound, and no evident mass was identified in the left adnexal region. Contemporaneous endocrine and tumor-marker results were not available; accordingly, this assessment should be interpreted as clinical and ultrasonographic follow-up rather than biochemical confirmation of remission. The follow-up remains too short to draw conclusions about long-term oncologic safety, contralateral ovarian risk, or reproductive outcomes.

## 4. Limitations

This report has several limitations. First, it describes a single patient and cannot establish disease-specific CEUS criteria. Second, the exact histopathological substrate of the focal microcystic spaces seen on ultrasound could not be demonstrated. Third, the laparoscopic specimen was fragmented, limiting precise pathological measurement of tumor size. Fourth, no tumor-specific testing for Y-derived material was documented; therefore, a 46,XX peripheral blood karyotype does not completely exclude very-low-level Y-chromosome mosaicism. Fifth, the contemporaneous MDT record did not itemize the adverse prognostic features or specify the chemotherapy regimen that would have been used if treatment had been selected. Sixth, contemporaneous serum endocrine and tumor-marker results were unavailable at the most recent 6.5-month follow-up. Finally, follow-up remains short for assessing recurrence, contralateral ovarian risk, and reproductive outcomes.

## 5. Conclusions

This case demonstrates how a histologically heterogeneous ovarian tumor can generate apparently discordant endocrine, biochemical, and imaging findings. Severe hyperandrogenism, progressive virilization, and marked vascularity predominantly suggested an androgen-producing sex cord-stromal tumor, whereas β-hCG elevation and CT findings raised concern for an additional germ-cell or trophoblastic component. Frozen section favored a steroid cell tumor but specifically did not exclude a focal germ-cell component. Final pathology unified these observations by demonstrating dissecting gonadoblastoma with dysgerminoma transformation and trophoblastic differentiation, combined with a Leydig cell tumor.

The central diagnostic lesson is that discordance itself can be informative. In an androgen-secreting ovarian tumor, unexplained β-hCG elevation should prompt consideration of an occult germ-cell or trophoblastic component. CEUS is useful for characterizing the vascular phenotype, but its enhancement pattern should be integrated with tumor markers, cross-sectional imaging, and pathology rather than interpreted as a histology-specific signature.

## Figures and Tables

**Figure 1:**
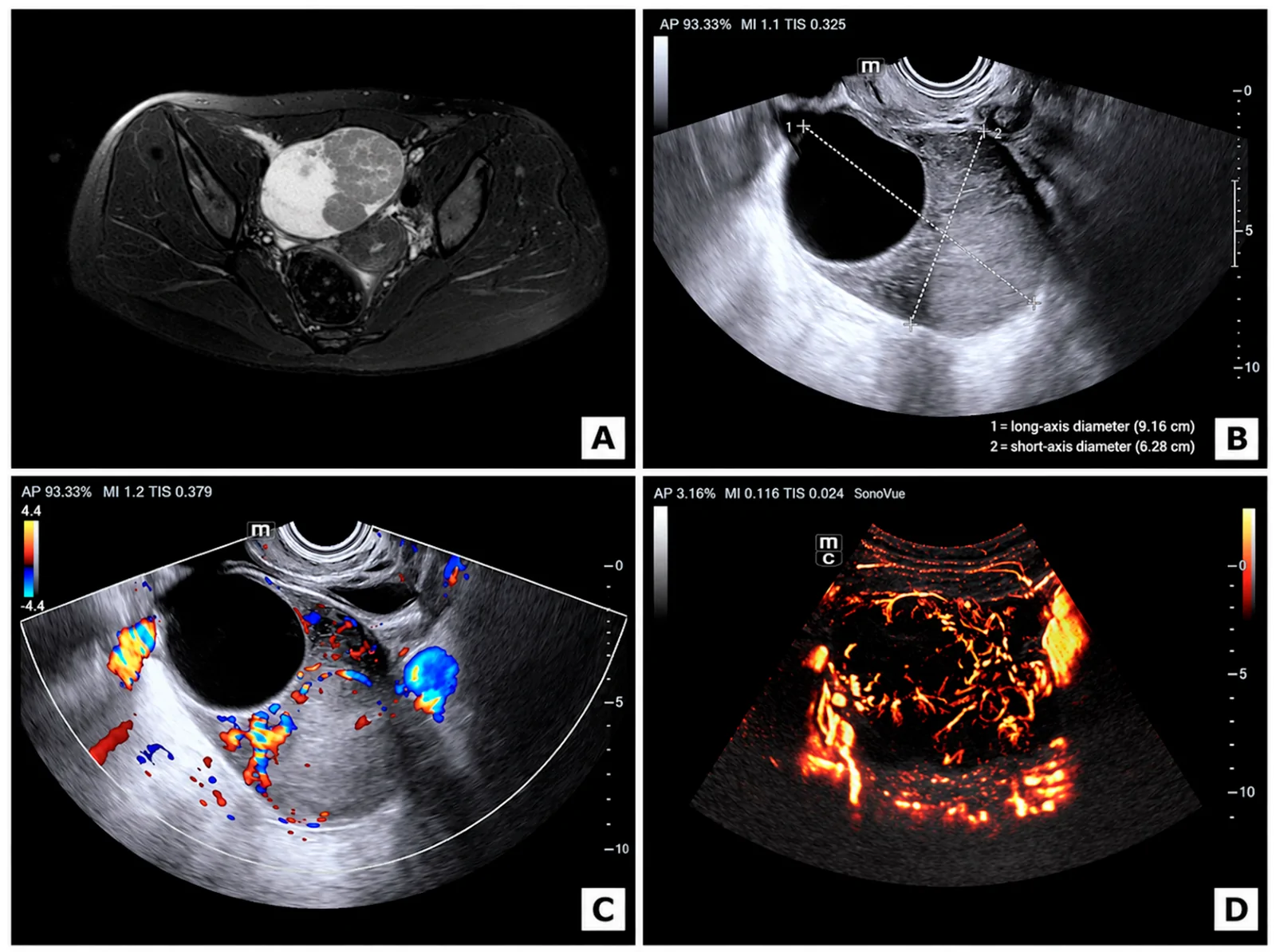
Preoperative multimodal imaging of the left adnexal mass. (**A**) Axial pelvic MR image showing the cystic-solid left ovarian mass. (**B**) Transrectal gray-scale ultrasound showing a well-circumscribed cystic-solid mass. (**C**) Color Doppler imaging showing internal vascular signals. (**D**) CEUS demonstrating heterogeneous hyperperfusion with rich intratumoral microvascularity. In panel (**B**), the dashed caliper lines represent the original lesion measurements.

**Figure 2:**
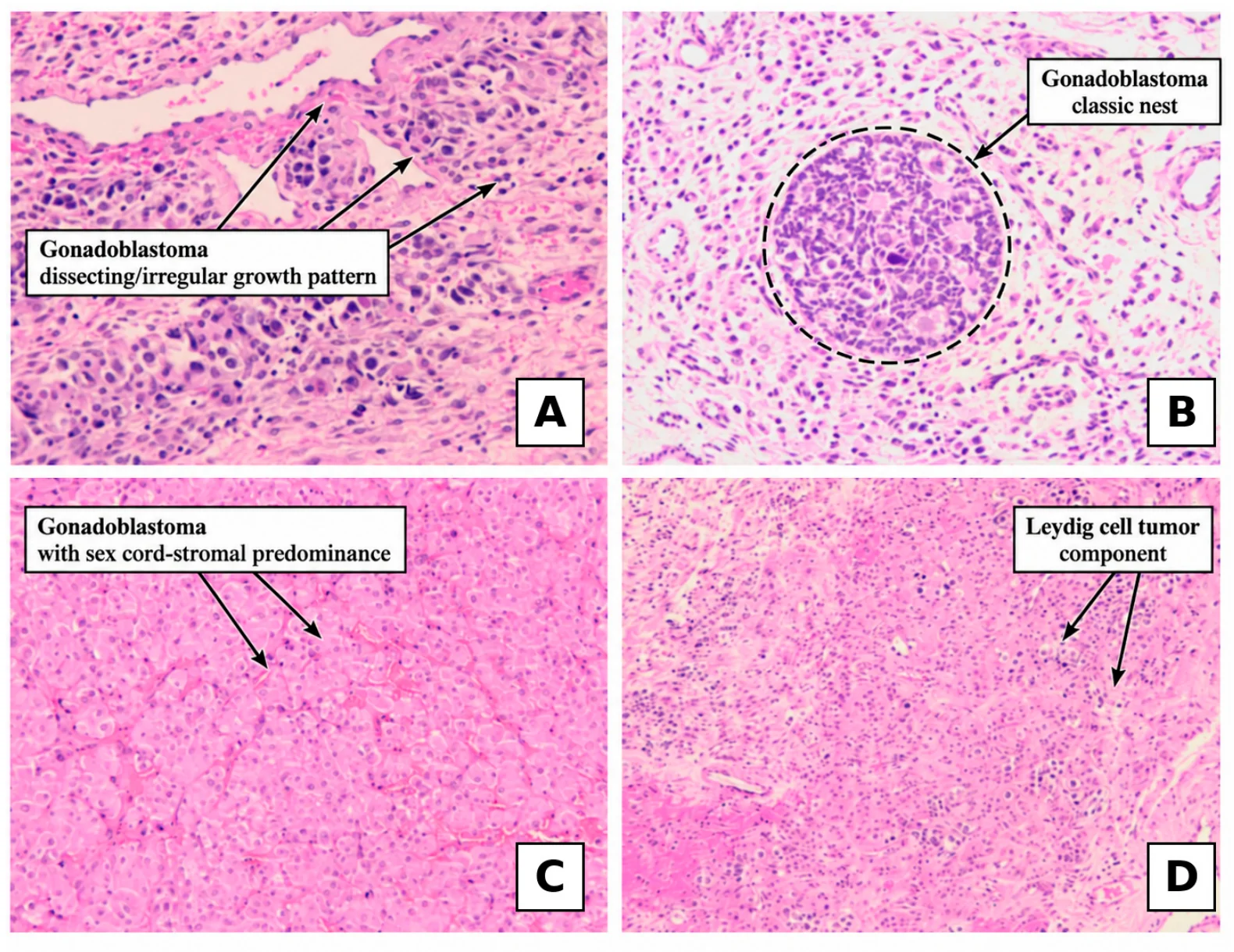
Selected histopathologic findings of the ovarian tumor (hematoxylin and eosin staining). (**A**) Dissecting gonadoblastoma showing irregular/dissecting architecture. (**B**) Classic gonadoblastoma nest composed of admixed germ cells and sex cord elements. (**C**) Area of gonadoblastoma with predominance of the sex cord-stromal component. (**D**) Leydig cell tumor component composed of polygonal cells with abundant eosinophilic cytoplasm. The illustrated panels represent selected morphologic components; dysgerminoma transformation and trophoblastic differentiation were established on complete histopathologic evaluation but are not specifically illustrated in this figure. Scale: 100 μm.

**Table 1:** Longitudinal clinical, biochemical, and imaging course.

Time Point	Clinical/Imaging Findings	Testosterone	β-hCG
September 2025	Progressive acne and voice deepening; left ovarian cystic lesion 4.1 × 3.8 × 2.9 cm	18.7 nmol/L	Not available
23 October 2025	Persistent menstrual disturbance despite hormonal treatment	42 nmol/L	Not available
19–24 December 2025	Rapidly enlarged ~9 cm cystic-solid left adnexal mass; secondary amenorrhea; marked virilization	>52 nmol/L; >16,000 pg/mL on detailed panel	192.0 IU/L
POD 1	After left salpingo-oophorectomy	28.1 nmol/L	80.4 IU/L
January 2026	Spontaneous menstruation resumed; androgen profile markedly normalized	104.7 pg/mL	<0.2 IU/L
7 July 2026	Clinically well; menstruation maintained; right ovary normal; no evident mass identified in the left adnexal region on ultrasound	No contemporaneous result available	No contemporaneous result available

Abbreviations: β-hCG, beta-human chorionic gonadotropin; POD, postoperative day.

**Table 2:** Diagnostic discordance and final clinicopathological interpretation.

Finding	Initial Implication	Final Clinicopathological Interpretation
Severe hyperandrogenism and virilization	Androgen-producing sex cord-stromal tumor, particularly a steroid cell or Leydig cell tumor	Leydig cell tumor component
Hypervascular MRI/CEUS phenotype	Sex cord-stromal tumor favored	Hypervascular phenotype without a one-to-one histologic correlate; not histology-specific
β-hCG 192.0 IU/L	Discordant with a pure androgen-producing tumor	Final pathology included trophoblastic differentiation; HCG positivity was present in a small number of cells
CT suspicion of mixed germ cell tumor	Possible germ-cell component	Gonadoblastoma with dysgerminoma transformation
Frozen section: steroid cell tumor; germ-cell component not excluded	Steroid-cell phenotype with a possible additional germ-cell component	Dissecting gonadoblastoma with dysgerminoma transformation and Leydig cell tumor

## Data Availability

The data supporting this case report are contained within the article. Additional de-identified clinical information may be available from the corresponding author upon reasonable request, subject to institutional and ethical restrictions.

## Supplementary Materials

The following supporting information can be downloaded at: https://www.mdpi.com/article/10.3390/diagnostics16193223/s1. Supplementary File S1: CARE Checklist.
