# Supplementary material for: Diagnostic Discordance in a 46,XX Ovarian Dissecting Gonadoblastoma with Dysgerminoma Transformation and Leydig Cell Tumor: Hyperandrogenism, β-hCG Elevation, and CEUS Findings
Source: Diagnostics (Basel). 2026 Oct 5;16(19):3223. doi: 10.3390/diagnostics16193223 (PMC13664418; doi:10.3390/diagnostics16193223)
Supplement: Supplementary file 1 [file diagnostics-16-03223-s001.zip › Supplementary_File_S1_CARE_Checklist.pdf]

Supplementary File S1. CARE Checklist

Case report: Diagnostic Discordance in a 46,XX Ovarian Mixed Germ Cell–Sex Cord-Stromal Tumor

CARE item	Topic	How addressed in this manuscript	Location
1	Title	The title identifies the report as a diagnostic case and specifies the principal condition and diagnostic features.	Title
2	Keywords	Key diagnostic terms are provided.	Keywords
3	Abstract	The abstract summarizes the presenting problem, diagnostic assessment, final diagnosis, intervention, follow-up, and take-home message.	Abstract
4	Introduction	The rarity and diagnostic challenge of ovarian mixed germ cell–sex cord-stromal tumors are summarized, with the rationale for reporting diagnostic discordance.	Section 1
5	Patient information	De-identified demographic information, menstrual history, endocrine manifestations, relevant treatment history, and family history are described.	Section 2.1
6	Clinical findings	Progressive virilization, acne, voice deepening, amenorrhea, and laboratory findings are reported.	Section 2.1
7	Timeline	The longitudinal clinical, biochemical, and imaging course is summarized.	Table 1
8	Diagnostic assessment	Endocrine testing, MRI, ultrasound/CEUS, CT, frozen section, permanent pathology, immunohistochemistry, FISH, and karyotyping are described; diagnostic uncertainty and discordance are discussed.	Sections 2.1–2.3; Sections 3.1–3.4
9	Therapeutic intervention	Fertility-sparing staging surgery and postoperative multidisciplinary discussion of surveillance versus chemotherapy are described.	Sections 2.3–2.4; Section 3.5
10	Follow-up and outcomes	Postoperative β-hCG and androgen decline, resumption of menstruation, genetic consultation, and 6.5-month ultrasound follow-up are reported.	Section 2.4; Table 1
11	Discussion	Strengths, diagnostic interpretation, relevant literature, limitations of CEUS, genetic context, management uncertainty, and report limitations are discussed.	Sections 3 and 4
12	Patient perspective	A formal patient-perspective statement was not collected and is therefore not reported.	Not available
13	Informed consent	Written informed consent for publication of the case and accompanying clinical, imaging, and pathological materials was obtained.	Informed Consent Statement

Note: This checklist maps the CARE reporting domains to the submitted manuscript. It is intended as a submission aid and does not add clinical information beyond the manuscript.
